# Information Theory and Symbolic Analysis: Theory and Applications

**DOI:** 10.3390/e23101361

**Published:** 2021-10-19

**Authors:** Mariano Matilla-García, Manuel Ruiz Marín

**Affiliations:** 1 Facultad de Económicas y Empresariales, Universidad Nacional de Educación a Distancia (UNED), 28040 Madrid, Spain; 2 Departamento Métodos Cuantitativos, Ciencias Jurídicas y Lenguas Modernas, Universidad Politécnica de Cartagena, 30201 Cartagena, Spain; manuel.ruiz@upct.es

Symbolic analysis has been developed and used successfully in very diverse fields. In recent literature, the contributions of symbolic analysis to the study of complex dynamics and network structures are easy to find, especially those based on symbolic entropy measures (such as permutation entropy) and the symbolic correlation integral (connected with Renyi and Tsallis entropies). Other notable studies include contributions that symbolic analysis has made to recurrence quantification analysis and its use in analyzing massive data.

The scientific fields applying symbolic analysis and its related information-theory concepts are quite varied. Thus, for example, we find applications in the fields of economics (in particular in finance and risk), geography and geolocation, and health, among others.

This Special Issue (SI) brings together contributions from researchers working in symbolic analysis, complex dynamics, and information theory, from both theoretical and applied perspectives. The original research papers presented in this issue contain enough reviewing material for those readers potentially interested in the corresponding lines of research. The SI includes applications of symbolic analysis and its entropy measures associated with the study of time series, spatial processes, and complex networks. As readers will see, great effort has been made to provide papers with real-world data.

A common factor of the seven published papers is that all of them make a theoretical contribution and then go on to illustrate their methods with a satisfying set of applications. Interestingly, almost all the contributors have decided to show the utility of their novel methods through health applications. Certainly, the SI explicitly indicated that applications to health were welcome but were by no means required. In general, health applications have been attractive to many researchers given the worldwide health circumstances in which we are living. Accordingly, this SI also includes the use of symbolic analysis tools for certain aspects of COVID-19.

C. Bandt [1] puts forward an entropy-based concentration coefficient C that avoids the limitations of other classical concentration measures such as Gini’s index. The limitations of well-known measures are made evident by the fact that some data, such as COVID-19 data on cases and deaths, might reflect the more fragmented spatial distribution of infections. Naturally, these new concentration measures can be used for other variables that can be sampled in this way: air and water pollution, precipitation, wealth, crime, etc. Interesting results are found when the C coeffient is applied to COVID-19 data coming from German counties.

Three papers investigating applications to neurological diseases provide intriguing results. Morales et al.’s paper [2] shows a method based purely on permutation entropy that is used to study the dynamics of epileptic brain activity using real EEG signals. The empirical results suggest that epileptic patients’ EEG signals reflect chaotic dynamics. Furthermore, the results show the power of the tools by distinguishing between healthy and epileptic subjects.

Cuesta-Frau et al. [3] contribute to the challenge of massively and semiautomatically monitoring and controlling bipolar disorder (BD), a widespread illness with huge social and economic impact. The authors study the feasibility of a BD episode classification analysis by using entropy measures. The task was a challenge since actigraphy records are highly non-stationary and corrupted with artifacts. The method is based on a quantification measure, Slope Entropy, which outperforms the most common entropy measures used in biomedical time series. The results confirm the feasibility of the approach, since the three states present in BD, depression, mania, and remission, can be clearly distinguished.

The use of symbolic patterns applied to neural activity is also found in the interesting paper by Masoliver and Masoller [4], where the way sensory neurons detect and transmit a weak external stimulus is studied. This is done by utilizing a well-established simulation model. The authors consider a sub-threshold stimulus; i.e., the stimulus is below the threshold triggering action potentials (spikes). However, in the presence of noise, the neuron that perceives the stimulus fires a sequence of action potentials (a spike train) that carries the information of the stimulus. The results suggest that sensory neurons can exploit the presence of neural noise to fire spike trains where the information of a subthreshold stimulus is encoded in over-expressed and/or under-expressed symbolic patterns.

The last three papers in this SI are related to the fields of networks, connections, and collaboration. The paper written by Nakayama [5] and collaborators is somewhat related to brain activity. By using recurrent symbolic tools, the authors designed an experiment in which pairs of players collaboratively created music in rhythmic improvisation. Rhythmic patterns and collaborative processes were investigated through symbolic-recurrence quantification the information theory applied to the time series of the sound created by the players. When the authors moved to the real data coming from the collaborative rhythmic improvisation, they identified features of improvised music and elucidated the underlying processes of collaboration. The results have important implications for those doing creative work in a collaborative environment. This paper has interested many readers according to the editorial information.

M. Makeienko’s paper [6] deals with house pricing formation. She studies the geographical components that might explain variations in real estate. To this end, she uses a symbol-based spatial test to confirm the nature of spatial dependence conditioned by the other covariates that usually explain the price of homes. She shows that models that merely incorporate space coordinates might be sufficient to capture space dependence. Hedonic models for Baltimore, Boston and Toledo housing price data sets are revisited, studied (with the new proposed procedures), and compared with standard spatial econometric methodologies.

Finally, Stephanovic et al. [7] are concerned with technology services and their quality. How can a model forecast a targeted value for Quality of Service (QoS) in a changing physical channel? In this paper, the nature of the data is very different from the other papers in the SI, but the symbol-based tools are valuable. The authors use a symbolic encapsulation point 5G (SEP5G) tool to improve and promote further extensions of the current, general 5G channel.
